# Multimorbidity and frailty are associated with poorer SARS-CoV-2-related outcomes: systematic review of population-based studies

**DOI:** 10.1007/s40520-023-02685-4

**Published:** 2024-02-14

**Authors:** Tatjana T. Makovski, Jinane Ghattas, Stéphanie Monnier-Besnard, Lisa Cavillot, Monika Ambrožová, Barbora Vašinová, Rodrigo Feteira-Santos, Peter Bezzegh, Felipe Ponce Bollmann, James Cottam, Romana Haneef, Brecht Devleesschauwer, Niko Speybroeck, Paulo Jorge Nogueira, Maria João Forjaz, Joël Coste, Laure Carcaillon-Bentata

**Affiliations:** 1 Department of Non-Communicable Diseases and Injuries, French Public Health Agency (Santé publique France), Saint–Maurice, France; 2 Institute of Health and Society (IRSS), Université catholique de Louvain, Brussels, Belgium; 3 Department of Epidemiology and Public Health, Sciensano, Brussels, Belgium; 4 National screening centre, Institute of Health Information and Statistics of the Czech Republic, Prague, Czech Republic; 5 Institute of Biostatistics and Analyses, Faculty of Medicine, Masaryk University, Brno, Czech Republic; 6 Área Disciplinar Autónoma de Bioestatística, Faculdade de Medicina, Universidade de Lisboa, Lisbon, Portugal; 7 Laboratório Associado TERRA, Instituto de Saúde Ambiental, Faculdade de Medicina, Universidade de Lisboa, Lisbon, Portugal; 8 Directorate for Project Management, National Directorate General for Hospitals, Budapest, Hungary; 9 Universidad Nacional de Educación a Distancia, Madrid, Spain; 10 Institute of Tropical Medicine, Antwerp, Belgium; 11 Department of Translational Physiology, Infectiology and Public Health, Ghent University, Merelbeke, Belgium; 12 Centro de Investigação Em Saúde Pública, Escola Nacional de Saúde Pública, ENSP, CISP, Comprehensive Health Research Center, CHRC, Universidade NOVA de Lisboa, Lisbon, Portugal; 13 CIDNUR—Centro de Investigação, Inovação e Desenvolvimento Em Enfermagem de Lisboa Escola Superior de Enfermagem de Lisboa, Avenida Professor Egas Moniz, 1600-190 Lisbon, Portugal; 14 National Center of Epidemiology, Instituto de Salud Carlos III, RICAPPS, Madrid, Spain

**Keywords:** COVID-19, SARS-CoV-2, Multimorbidity, Multiple chronic conditions, Frailty

## Abstract

**Background:**

Estimating the risks and impacts of COVID-19 for different health groups at the population level is essential for orienting public health measures. Adopting a population-based approach, we conducted a systematic review to explore: (1) the etiological role of multimorbidity and frailty in developing SARS-CoV-2 infection and COVID-19-related short-term outcomes; and (2) the prognostic role of multimorbidity and frailty in developing short- and long-term outcomes. This review presents the state of the evidence in the early years of the pandemic. It was conducted within the European Union Horizon 2020 program (No: 101018317); Prospero registration: CRD42021249444.

**Methods:**

PubMed, Embase, World Health Organisation COVID-19 Global literature on coronavirus disease, and PsycINFO were searched between January 2020 and 7 April 2021 for multimorbidity and 1 February 2022 for frailty. Quantitative peer-reviewed studies published in English with population-representative samples and validated multimorbidity and frailty tools were considered.

**Results:**

Overall, 9,701 records were screened by title/abstract and 267 with full text. Finally, 14 studies were retained for multimorbidity (etiological role, *n* = 2; prognostic, *n* = 13) and 5 for frailty (etiological role, *n* = 2; prognostic, *n* = 4). Only short-term outcomes, mainly mortality, were identified. An elevated likelihood of poorer outcomes was associated with an increasing number of diseases, a higher Charlson Comorbidity Index, different disease combinations, and an increasing frailty level.

**Discussion:**

Future studies, which include the effects of recent virus variants, repeated exposure and vaccination, will be useful for comparing the possible evolution of the associations observed in the earlier waves.

**Supplementary Information:**

The online version contains supplementary material available at 10.1007/s40520-023-02685-4.

## Introduction

Since the start of the COVID-19 pandemic in 2019, understanding the impact of SARS-CoV-2 infection on population health has become more coherent. Much evidence has emerged, among others, for different age groups [1–3], patient groups such as those with immunodeficiency or kidney transplant [4, 5] or population groups such as health care workers or pregnant women [6, 7] or on the specific effects of COVID-19 such as mental health consequences, though predominantly among non-infected individuals [8, 9].

Advanced age is repeatedly mentioned as an independent risk factor for death and other adverse outcomes among infected individuals [10, 11]. Findings from China in the early period of the pandemic showed an increase in the COVID-19-related case fatality ratio with age, of e.g., 0.4% or less in 40-year olds or younger, 1.3% among individuals in their 50s, 3.6% among individuals in their 60s, 8% among 70-year olds, and 14.8% in those aged 80 years or older [12]. Furthermore, older age is likely to be accompanied by one or multiple chronic diseases, commonly called multimorbidity [13], or the accumulation of deficits in different body systems, known as frailty [14]. In fact, 55 to 98% individuals over the age of 60 live with multiple chronic conditions [15], while multidimensional frailty prevalence ranges from 13.3% in population-based settings to 51.5% in nursing homes [16]. Moreover, it is worth recalling that the number of elderly people have been increasing worldwide for decades. The European Union, for example, had 90.5 million citizens aged 65 years and over in 2019 with projections of 129.8 million by 2050; the age group of 85 and older is growing at the fastest pace, with a projected increase of approximately 113.9% by 2050 [17]. This demographic change is observed globally, thus highlighting the associated challenges such as multimorbidity and frailty.

The risks of poor COVID-19-related outcomes have been well documented in patients with single chronic conditions. Studies have underlined hypertension, diabetes, cardiovascular diseases and chronic pulmonary or kidney diseases as important factors contributing to the increased in-hospital case fatality rate [18]. However, despite growing challenges associated with disease accumulation in rapidly expanding ageing populations [19], exploring the interplay between different numbers and combinations of health conditions and their impact on COVID-19-related outcomes has not yet been sufficiently explored [18].

Both multimorbidity and frailty may interfere with the physiological processes of individuals, thus making them more susceptible to infection or adverse outcomes in COVID-19 illness. Evidence about their role in the COVID-19 pandemic identified increased mortality risks and intensive care unit (ICU) admissions in patients with frailty and multimorbidity [20, 21]. However, these findings are primarily based on hospital settings, which precludes the generalisation of the strength of these associations to the general population. Assessing and stratifying these risks at the population level is thus essential for informing public health decision-making and actions [22].

Using a population-based approach, we conducted a systematic literature review with two objectives: (1) to evaluate the etiological role of multimorbidity and frailty in developing SARS-CoV-2 infection and COVID-19-related short-term outcomes; and (2) to investigate the prognostic role of multimorbidity and frailty in developing short- and long-term COVID-19 outcomes. This paper reports all the outcomes identified in the literature meeting all the study criteria.

This study was part of a broader analytical work funded by the European Union’s (EU) Horizon 2020 research and innovation program under the grant agreement no. 101018317, which considered both biomedical and socioeconomic factors [23, 24]. This paper only focuses on biomedical factors, namely multimorbidity and frailty.

## Methods

The study was conducted according to the Preferred Reporting Items for Systematic Review and Meta-Analysis Protocols (PRISMA-P) 2015 [25]. The protocol was registered in the PROSPERO registry for systematic review protocols (no. CRD42021249444) and previously described [23]. Thus, only the main methodological points are outlined below.

### Study design and search strategy

We applied a population-based approach and considered only studies conducted on well-defined populations of *individuals residing in a defined geographic region in a given time period* [26]. The analysis could include all individuals or a random population sample, the setting could be community- and/or hospital-based and the design could be based on case–control, cross-sectional or prospective and retrospective cohort studies. The key point is that the individuals included in the studies are *representative* of all individuals in the well-defined population [27].

All types of observational studies with comparative groups were eligible, either etiological: considering risk factors, believed to be related to the probability of an individual of the population developing SARS-CoV-2 infection or COVID-19-related health event; or prognostic: considering prognostic factors, believed to be related to the probability of an individual *with SARS-CoV-2 infection or COVID-19* of the population developing a certain outcome [28]. Interventions, clinical trials, case and qualitative studies were excluded.

We defined multimorbidity as two or more conditions in an individual [13] and included all reports of the association between any two or more conditions and the outcomes of interest. In the literature, multimorbidity is usually assessed by measurements such as disease count, comorbidity or multimorbidity indices or disease combinations (i.e., disease clusters or patterns). These measurements of multimorbidity were considered in the review.

Similarly, we retained studies on frailty which referred to one of the three most commonly used scales, the phenotypic approach by Fried et al. 2001 [29], the frailty index [30] and the clinical frailty scale [31] from the deficit accumulation approach by Rockwood.

In terms of outcomes, the following was considered: short-term outcomes could be SARS-CoV-2 infection confirmed with a test or the information on COVID-19 diagnosis retrieved from hospital records or medico-administrative data, patient hospitalisation, mechanical ventilation, and mortality linked to COVID-19; long-term outcomes could be quality of life, mental health, or functional decline, identified through validated scales.

The systematic search strategy included variations of key terms for multimorbidity, frailty, socioeconomic characteristics, COVID-19 and study design. Supplementary material 1 presents details about the search strategy.

### Data sources

The following databases were searched: PubMed, Embase, PsycINFO and World Health Organisation COVID-19 Global literature on coronavirus disease [32].

The initial search was performed on 7 April 2021 to identify literature published from January 2020 onwards. Only peer-reviewed literature published in English was considered. A search update was performed for frailty alone on 1 February 2022 due to the scarcity of evidence identified in the initial search. Supplementary material 2 describes the search strategy used for this update.

### Study selection

Screening, data extraction and study quality assessment were performed in pairs. Due to the broader research objectives of the consortium (see Introduction), the record screening phase was performed jointly for biomedical (frailty and multimorbidity) and socioeconomic risk factors. TM and JG acted as the first reviewers and shared the literature during this screening phase. They remained as the first reviewers at the later stage for their respective research topics of biomedical or socioeconomic factors. Eleven colleagues acted as second reviewers (LCB, SMB, LC, RH, RFS, FPB, JC, MA, BV, PB, PJN); five for biomedical part alone. Both teams referred to a third party in the case of disagreements (JCo).

The exclusion criteria for the record screening phase are presented in List 1.

- List 1: Reasons for exclusion from the systematic review (title/abstract or record screening phase) and number of excluded studies

**Table:** 

Criteria for exclusion	Number of excluded studies
1 = Language other than English	7
2 = Not original research (e.g., editorial, protocol) or no original results	597
3 = Unrelated topic (e.g., studies on individual diseases, outside the review scope)	8,078
4 = Not a population-based study	534
5 = Subpopulation (e.g., pregnant women, health care workers, students)	74

The report screening phase was conducted using the following exclusion criteria (List 2).

- List 2: Reasons for exclusion from the systematic review (full-text or report screening phase) and number of excluded studies

**Table:** 

Reasons for exclusion	Number of excluded studies
1 = Not a population-based study	90
2 = Unclear SARS-CoV-2 infection diagnosis*	not assigned
3 = Study does not consider people with frailty or multimorbidity	88
4 = Unclear outcome measurement tool*	not assigned
5 = Unclear risk factor measurement tool*	not assigned
6 = Subpopulation (e.g., pregnant women, health care workers, students)	not assigned
7 = Not original research (e.g., editorial, protocol, review, conference abstract, grey literature), no original results or not peer-reviewed	28
8 = Identical or almost identical population considered in another study with the same outcome	4
9 = Clinical trial or intervention study	not assigned
10 = Qualitative study	not assigned
11 = Descriptive study, absence of comparative group and/or no measurement of the association of interest	16
12 = Other (explain)**	27

*even after contacting the study authors.

**full text not found, research question beyond the review scope, ecological study, case series, etc.

The authors were contacted to provide additional details for the studies with missing information. The reference lists of the selected studies were explored (snowballing) to identify additional potential evidence.

### Data extraction

For each study, information was extracted into customised tables for each study objective and each outcome and separately for multimorbidity and frailty. The information concerned the study sample characteristics, exposure and outcome details as well as association measures.

### Quality and risk of bias assessment

All studies retained for the final synthesis were assessed for quality using the Newcastle–Ottawa scales [33–35]. Higher scores indicated better study quality, with a maximum score of 9 points for cohort studies and 10 points for cross-sectional studies.

## Results

**Fig.1:**
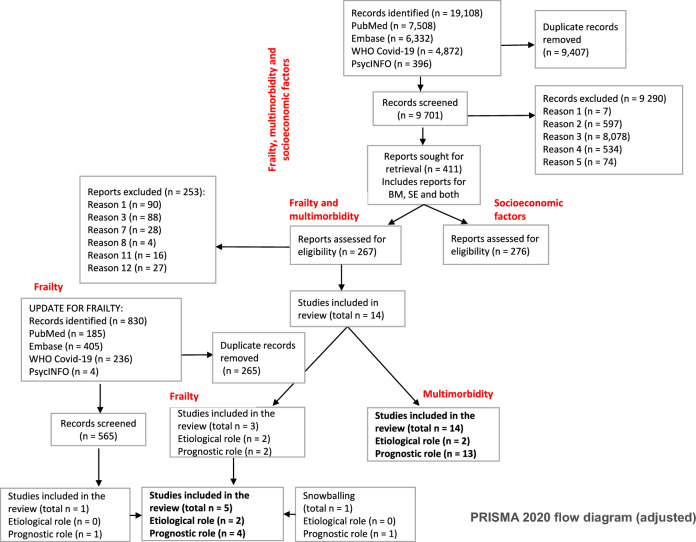
PRISMA 2020 flow diagram for new systematic reviews, which included searches of databases and registers only (adjusted)

A total of 9,701 records were screened for title and abstract in relation to socioeconomic and biomedical risk factors (Fig. 1), with 411 full-text studies being assessed for inclusion. Of these publications, 267 focused on the biomedical risk factors of multimorbidity and frailty. The search update conducted on frailty on 1 February 2022 retrieved 830 articles: 565 were screened for title and abstract, while only one article was retained. Snowballing for multimorbidity and frailty identified one additional article for frailty.

Finally, 14 studies were retained for data synthesis for multimorbidity (etiological role, *n* = 2; prognostic role, *n* = 13) and five for frailty (etiological role, *n* = 2; prognostic role, *n* = 4). The outcomes considered for prognosis were SARS-CoV-2 infection, hospitalisation (including ICU admission/ventilation) and mortality, and the maximum follow-up time was 9 months [36].

Details about the reasons for study exclusion are provided in Lists 1 and 2, Fig. 1 and Supplementary material 3.

The average disagreement between the first and second reviewers during the first screening phase for both socioeconomic and biomedical risk factors was 2.1%. The disagreement between two reviewers for the biomedical full-text (report) reading stage was 10%. The third reviewer was consulted 24 times, mainly to provide advice when the representativeness of the study sample was not clear.

Five authors were contacted for additional information, with two of them providing usable information.

The findings are further described below for multimorbidity and frailty separately.

### Multimorbidity

#### Study characteristics

The study criteria were met by 14 studies relating to multimorbidity [36–49]. Two studies described the etiological role of multimorbidity [36, 37], while 13 explored its prognostic role [36, 38–49]. The study of Mak et al. [36] complied with both study objectives. Ten studies were cohorts, while four were cross-sectional. The majority of the studies were conducted in high-income countries, with only a few in mid-income settings. Overall, study quality was good; seven studies scored 8 points and one scored 7 points; two cohort studies and three cross-sectional studies scored the maximum of 9 and 10 points, respectively (Table 1 and Supplementary material 4). Five studies were conducted in community settings, four among hospitalised patients, four among outpatients and hospitalised patients and one in the ICU. Only two studies defined multimorbidity, as two or more comorbidities [43] or two or more chronic diseases [38]. The other studies did not specify the definition but instead presented the associations between increasing disease burden (e.g., number of diseases, Charlson Comorbidity Index) and COVID-19 health outcomes. Information on chronic conditions was mainly retrieved from medical records, although the use of a medical coding system was rarely reported. The maximum number of included diseases was 23, while the minimum was 7; only one study did not report the conditions under consideration. COVID-19 diagnosis was usually confirmed by a polymerase chain reaction (PCR) test.

**Table 1:** Characteristics of the studies reporting on multimorbidity and frailty in population-representative samples (etiological and prognostic role)

Studies describing the etiological role											
*Authors (Country)*	*Study design* *Study setting* *Study inclusion*	*Study quality score*^*a*^	*Study inclusion and/or follow-up period*	*Outcome*	*Outcome measure*	*Study participants included in the analysis and available sociodemographic characteristics*	*Multimorbidity (measurement, number of conditions and source of information; prevalence where available)*	*List of conditions considered in the study*	*Frailty (definition, measurement, number of conditions, source of information; prevalence where available)*	*Association metrics between multimorbidity or frailty and the outcome; adjusted analyses (yes/no/both)*	*Total number (%) of people for which the outcome occurred*
*Mak *et al*. 2021 (England)*	Cohort Community study (UK biobank) Inclusion: UK biobank subjects aged 49–86 years and alive on 1 March 2020	8	Inclusion: 1 March 2020 Follow-up: 1 March to 30 November 2020	1) *Infection* 2) *Hospitalisation* 3) *Mortality*	1) RT-PCR SARS-CoV-2 positive test 2) Hospital records of Covid-19 (ICD-10 = U.07) 3) Covid-19 cause of death on death certificate	*N* = 410,199 Age, mean (SD) = 67.6 (8.1); females: 55.1%; ethnicity: white (94.3%), Asian (2.4%), black (1.8%), others (1.6%); education: low (16%), intermediate (51%), high (33%); Townsend deprivation quintile: 1 (least deprived) (20.1%), 2 (20.4%), 3 (20.2%), 4 (20%), 5 (most deprived (19.2%); income: < £18,000 (21.7%), £18,000–30,999 (25.3%), £31,000–51,999 (26.4%) ≥ £52,000 (26.6%)	Measured by CCI; 17 conditions retrieved from hospital records (ICD-10 codes)	Myocardial infarction, congestive heart failure, peripheral vascular disease, cerebral vascular disease, dementia, pulmonary disease, connective tissue disorder, peptic ulcer, liver disease, diabetes, diabetes complications, paraplegia, renal disease, cancer, metastatic cancer, severe lever disease, acquired immune deficiency syndrome	Hospital frailty risk score (HFRS) Information retrieved from electronic medical records HFRS bands: Low risk: 402,832 (98.2%) Intermediate risk: 7,120 (1.7%) High risk: 247 (0.1%)	OR (95% CI) Adjusted	1) Infection *N* = 7,590 (1.85%) 2) Hospitalisation *N* = 2,812 (0.69%) 3) Mortality *N* = 514 (0.1%)
*Izurieta *et al*. 2020 (US)*	Retrospective cohort Community study (US Medicare beneficiaries) Inclusion: Medicare beneficiaries aged ≥ 65 years, 1 April to 8 May 2020	8	Follow-up: 1 April to 8 May 2020	1) *Hospitalisation* 2) *Mortality*	1) Hospital records of Covid-19 (ICD-10 = U.071) 2) All death with prior discharge diagnosis of Covid-19 or ≥ 2 professional claims with Covid-19 within 21 days of death or 1 if immediately before death	*N* = 24,367,476 Median age = 73; females: 55.6%	Measured by CCI; conditions retrieved from electronic medical records (ICD-10 codes) within 6 months prior to study CCI > 0: 32.6%	No details	Electronic frailty index Information retrieved from electronic medical records (ICD-10 codes) within 6 months prior to study Level 1: 85.2% Level 2: 11.3% Level 3: 2.7% Level 4: 0.7% Level 5: 0.1%	OR (95% CI) Adjusted	1) Hospitalisation *N* = 27,961 (0.11%) 2) Mortality *N* = 12,613 (0.05%)
Studies describing the prognostic role											
*First author (Country)*	*Study design* *Study setting* *Study inclusion*	*Study quality score*^*a*^	*Study inclusion and/or follow-up period*	*Covid-19 diagnosis for population selection*	*Outcome and outcome measure*	*Study participants included in the analysis and available sociodemographic characteristics*	*Multimorbidity (measurement, number of conditions and source of information; prevalence where available)*	*List of conditions considered in the study*	*Frailty (definition, measurement, number of conditions, source of information; prevalence where available)*	*Association metrics between multimorbidity or frailty and the outcome; adjusted analyses (yes/no/both)*	*Total number (%) of people for which the outcome occurred*
*Mak *et al*. 2021 (England)*	Retrospective cohort Inclusion: UK biobank subjects hospitalised for Covid-19 between 1 March and 30 November 2020	8	1 March to 30 November 2020	Inpatient: Positive RT-PCR SARS-CoV-2 test or hospital records of Covid-19 (ICD-10 = U.07) or died of COVID-19, defined as those with COVID-19 (ICD-10 code U07) as the primary or contributory cause of death	*Mortality* COVID-19 cause of death in death certificates	*N* = 2,812 Age, mean (± SD) = 69.2 (± 8.7); females: 45.1%; ethnicity: white (90.6%), Asian (4.2%), black (3.3%), others (1.9%); education: low (30.1%), intermediate (48.6%), high (21.3%); Townsend deprivation quintile: 1 (least deprived) (13.9%), 2 (16.3%), 3 (18%), 4 (21%), 5 (most deprived (30.8%); income: < £18,000 (36.9%), £18,000–30,999 (25.6%), £31,000–51,999 (21.5%) ≥ £52,000 (16.1%)	Measured by CCI; 17 conditions retrieved from hospital records (ICD-10 codes)	Myocardial infarction, congestive heart failure, peripheral vascular disease, cerebral vascular disease, dementia, pulmonary disease, connective tissue disorder, peptic ulcer, liver disease, diabetes, diabetes complications, paraplegia, renal disease, cancer, metastatic cancer, severe liver disease, acquired immune deficiency syndrome	Hospital frailty risk score (HFRS) Information retrieved from electronic medical records HFRS bands: Low risk: 2,153 (76.6%) Intermediate risk: 339 (12.1%) High risk: 320 (11.4%)	OR (95% CI) Adjusted	*N* = 417 (14.8%)
*Navaratnam *et al*. 2021 (England)*	Retrospective cohort Hospital-based study Inclusion: all patients ≥ 18 years hospitalised for COVID-19 between 1 March and 31 May 2020	9	1 March to 31 May 2020	Hospital records of Covid-19 diagnoses (ICD-10 = U.071, U.072)	*Mortality* In-hospital death as recorded by the Office for National Statistics	*N* = 91,541 Statistical analysis performed on *N* = 79,124 Age ≥ 80 = 34.8%; females 44.5; ethnicity: white (70.6%), Asian or Asian British (7.8%), Black or Black British (5.4%), mixed (0.81%), other ethnic groups (3.6%), missing data (11.8%); deprivation quintile: 1 (most deprived) (25.1%), 2 (22%), 3 (18.7%), 4 (16.9%), 5 (least deprived) (15.2%), missing data (2%)	Measured by CCI; 14 conditions retrieved from hospital records (ICD-10 codes) N conditions = *N* (%) population 0 = 25,120 (27.4) 1 = 23,579 (25.8) 2 = 15,244 (16.7) 3 = 10,832 (11.8) ≥ 4 = 16,766 (18.3) ≥ 2 conditions = 42,842 (54.1)	Myocardial infarction, congestive heart failure, peripheral vascular disease, cerebral vascular disease, dementia, pulmonary disease, connective tissue disorder, peptic ulcer, liver disease, diabetes and diabetes complications, paraplegia or hemiplegia, renal disease, cancer and metastatic cancer, severe liver disease, acquired immune deficiency syndrome	Hospital frailty risk score (HFRS) Information retrieved from electronic medical records HFRS bands: None: 34,658 Mild: 7,213 Moderate: 21,137 Severe: 28,533	OR (95% CI) Adjusted	*N* = 28,200 (30.8%)
*Hodgson *et al*. 2021 (Australia)*	Cohort Covid-19 patients admitted to ICU Between 6 March and 4 October 2020	9	Inclusion: 6 March to 4 October 2020 Follow-up: 6-months after inclusion	Positive RT-PCR for Sars-CoV-2 laboratory test	*Mortality or new disability* Death or new disability at 6 months Data source for death was not specified New disability was defined as an increase of ≥ 10% in WHODAS at 6 months from baseline	*N* = 160 Median (IQR) age: 62 (55–71); females: 39.4%; median (IQR) years of education: 14 (11–16)	Multimorbidity not assessed	Multimorbidity not assessed	Clinical frailty scale (CFS) Questionnaire at the time of ICU admission (physical function in the month preceding admission) Median (IQR) CFS: 3 (2–3)	OR (95% CI) Unadjusted and adjusted	Death *N* = 43 (26.9%) New disability *N* = 42 (38.9%) Combined outcome *N* = 85 (53.1%)
*Kundi *et al*. 2020 (Turkey)*	Cross-sectional study Hospital-based study Inclusion: all hospitalised patients ≥ 65 years from 11 March to 22 June 2020	9	Inclusion: data between 11 March and 22 June 2020	At least one positive RT-PCR test	1) *All-cause mortality* In-hospital death 2) *Intensive care unit* 3) *Invasive mechanical ventilation*	*N* = 18,234 Mean age (SD): 74.1 (7.4); females: 53.4%	Multimorbidity not assessed	Multimorbidity not assessed	Hospital frailty risk score (HFRS) Information retrieved from health records Low HFRS (< 5 points): *N* = 5,814 (31.9%) Intermediate HFRS (5–15 points): *N* = 9,619 (52.8%) High HFRS (> 15 points): *N* = 2,801 (15.4%) HFRS ≥ 5 defined as frail: *N* = 12,295 (67.4%) Mean HFRS (SD): 8.9 (7.0)	OR (95%CI) Adjusted	1) All-cause mortality 2) *N* = 3,315 (18.2%) 3) ICU 4) *N* = 4,510 (24.7%) 5) Invasive mechanical ventilation 6) *N* = 3,080 (16.9%)
*Haase *et al*. 2020 (Denmark)*	Cohort ICU patients Inclusion: first identified case to 19 May 2020	7	Follow-up: at least 28 days; median follow-up 79 days (range 28–96, IQR 71–84) among survivors	RT-PCR test	*Mortality* In-hospital death	*N* = 308 Median age (IQR): 68 (59–75); females: 26%	Measured by disease count; 10 conditions retrieved from electronic patient files *N* conditions = N people 0 = 90 1 = 85 2 = 76 3 = 40 4 = 10 5 = 6 6 = 1 ≥ 2 conditions = 133 (43%)	Hypertension, ischemic heart disease, heart failure, chronic pulmonary disease, chronic kidney disease, liver cirrhosis, diabetes, active cancer, hematologic malignancy, immunosuppressed	Frailty not assessed	Mortality rate Unadjusted	*N* = 118 (37%)
*Millán-Guerrero *et al*. 2020 (Mexico)*	Cohort Patients attending any health care facility (69.3% received ambulatory care, 30.7% hospitalised) Inclusion: 27 February to 1 July 2020	9	Follow-up: 3–4 weeks after diagnosis	PCR test	*Mortality* Final outcome (survival or death) based on administrative data	N = 231,772 Age range: 0- ≥ 80; females: 45.4%; population: indigenous (1.1%), non-indigenous (98.9%); poverty in municipality^1^: 0–20 (10.7%), 20.1–40 (53.8%), 40.1–60 (25.6%), > 60 (10.0%)	Measured by disease count; 8 conditions retrieved from administrative data	Hypertension, obesity, diabetes, smoking, asthma, cardiovascular disease, chronic kidney disease, chronic obstructive pulmonary disease	Frailty not assessed	HR (95% CI) Unadjusted and adjusted	*N* = 28,510 (12.3%)
*Reilev *et al*. 2020 (Denmark)*^*b*^	Cohort SARS-CoV-2 PCR positive cases (80% community-managed and 20% hospitalised) Inclusion: 27 February to 19 May 2020	8	Follow-up: 30 days	PCR test	*Mortality* All-cause mortality defined as deaths occurring from 2 days before the index date (date of the first positive PCR test) to 30 days after *Hospitalisation* Hospital admissions due to COVID-19 defined as continuous in-hospital stays with a duration of 12 h or longer occurring up to 14 days after the index date	*N* = 11,122 Age range 0 − ≥ 90; median age (IQR): 48 (33–62), varying from 44 years (IQR 30–56) among non-hospitalised cases to 82 years (IQR 75–88) among those who died; females: 58%	Measured by disease count; 17 conditions retrieved from administrative and health registries *N* conditions = *N* (%) population 0 = 6,034 (54) 1 = 2,462 (22) 2 = 1,140 (10) 3 = 691 (6.2) ≥ 4 = 795 (7.1) ≥ 2 conditions: 15% of community-managed cases, 56% of hospitalised cases, 79% of fatal cases	Chronic lung disease, hypertension, ischaemic heart disease, heart failure, atrial fibrillation, stroke, diabetes, dementia, any cancer, chronic liver disease, hospital-diagnosed kidney disease, alcohol abuse, substance abuse, major psychiatric disorder organ transplantation, medical overweight and obesity, rheumatoid arthritis/connective-tissue disease	Frailty not assessed	OR (95% CI) Unadjusted and adjusted	*Mortality* *N* = 577 (5.2%) *Hospitalisation* *N* = 2,254 (20%)
*Sousa *et al*. 2020 (Brazil)*	Cross-sectional study Population admitted to hospital or paediatric health care unit Inclusion: 1 January to 14 July 2020	9	Data gathered from 1 January to 14 July 2020	RT-PCR test	*Mortality* In-hospital death	*N* = 4,784 (2,570 Covid-19 positive patients; remaining, other SARI patients) Age range < 30 days: 19.99 years; females: 48%; non-white ethnicity: 59.7%	Measured by disease count; 10 conditions retrieved from medical records 0 = 1,584 (61.6%) 1 = 707 (27.5%) ≥ 2 = 279 (10.9%)	Down syndrome, diabetes mellitus, immunodepression, chronic cardiovascular diseases, chronic hepatic diseases, chronic neurological conditions, chronic kidney diseases, chronic hematologic diseases, asthma, chronic pneumopathies	Frailty not assessed	OR (95% CI) Unadjusted and adjusted	*N* = 353 (15.2%)
*Hernandez-Vasquez *et al*. 2020 (Mexico)*	Cross-sectional study Community study Inclusion: through 18 May 2020	10	Inclusion: data available ending 18 May 2020	RT-PCR test	*Mortality* Death in patients with COVID-19	*N* = 51,053 Age range: 0 − ≥ 85; mean age (± SD) 46.6 (± 15.8); females: 42.4%	Multimorbidity defined as the presence of ≥ 2 chronic comorbidities; measured by disease count; 8 conditions retrieved from medical records *N* conditions = *N* (%) population 0 = 27,667 (54.2) 1 = 13,652 (26.7) 2 = 6,518 (12.8) 3 = 2,490 (4.9) 4 = 572 (1.1) 5 = 109 (0.2) 6 = 24 (0.0) 7 = 7 (0.0) 8 = 14 (0.0) ≥ 2 conditions = 9,734 (19.1)	High blood pressure, diabetes, obesity, asthma, immunosuppression, other cardiovascular diseases, chronic obstructive pulmonary disease, chronic kidney disease	Frailty not assessed	OR (95% CI) Unadjusted and adjusted	*N* = 5,233 (10.3%)
*Cho *et al*. 2020 (South Korea)*^*b*^	Cohort Community study Inclusion: 1 February to 15 May 2020	8	Follow-up mean (± SD): 20.9 ± 13.1 (days)	COVID-19 patients defined as patients with diagnostic codes for COVID-19	*Mortality* Data did not include cause of death, although most deaths were caused by infection-related complications such as sepsis or acute respiratory distress syndrome *Mechanical ventilation* Mechanical ventilation and other outcomes were assessed after COVID-19 diagnosis	*N* = 7,327 Age mean (± SD): 47.0 (± 19.0); females: 59.5%	Measured by CCI; 17 conditions (part of CCI) retrieved from administrative data (ICD-10 codes)	Myocardial infarction, congestive heart failure, peripheral vascular disease, cerebrovascular disease, dementia, chronic pulmonary disease, rheumatologic disease, peptic ulcer disease, mild liver disease, diabetes mellitus without chronic complications, hemiplegia, renal disease, diabetes mellitus with chronic complications, any malignancy, moderate to severe liver disease, metastatic tumour and acquired immune deficiency syndrome	Frailty not assessed	HR (95% CI) Unadjusted and adjusted	*Mortality* *N* = 223 (3%) *Mechanical ventilation* *N* = 123 (1.7%)
*Argoty-Pantoja *et al*. 2021 (Mexico)*	Cohort Outpatients and hospitalised COVID-19 patients Inclusion: 27 February to 30 July 2020	8	Inclusion: 27 February to 30 July 2020	Positive diagnosis for SARS-CoV2 infection certified by Institute of Epidemiological Diagnosis and Reference	*Mortality* Fatality rate defined as the ratio of the number of deaths that occurred in the cohort study of confirmed COVID-19 cases, and the person-time at risk	*N* = 412,017 Age range: < 35 − ≥ 65; females: 46.8%; population: indigenous (1.1%), non-indigenous (98.9%)	Measured by disease combinations; 9 conditions considered, source of information not specified Disease combinations = *N* (%) population Obesity and hypertension = 13,814 (3.4) Diabetes and hypertension = 24,004 (5.8) Diabetes and obesity = 7,098 (1.7‬) Diabetes and obesity and hypertension = 11,346 (2.8)	Chronic obstructive pulmonary disease, asthma, immunosuppression, cardiovascular disease, chronic kidney disease, smoking, metabolic comorbidities (combined effect of diabetes, hypertension and obesity)	Frailty not assessed	HR (95% CI) Adjusted	*N* = 45,754 (11.1%)
*Ticinesi *et al*. 2021 (Italy)*	Cross-sectional study Hospital-based study Inclusion: Patient age ≥ 18 years hospitalised for COVID-19 between 28 February and 10 June 2020	10	Inclusion: 28 February to 10 June 2020	Presence of symptoms and radiological features compatible with COVID-19 pneumonia Sensitivity analysis with a subsample of individuals tested using RT-PCR SARS-CoV-2 test	*Mortality* In-hospital death	*N* = 1,264 Age range: 20–99; females: 43.8%	Multimorbidity defined as the presence of ≥ 2 chronic diseases; measured by disease count using a list of 23 diseases; information retrieved from clinical records Multimorbidity (≥ 2 diseases) *N* = 923 (73%)	Includes (but not all listed in the manuscript): hypertension, diabetes, obesity, heart failure, chronic kidney disease, chronic obstructive pulmonary disease, asthma, previous stroke, parkinsonism, dementia, epilepsy, cancer	Frailty not assessed	OR (95% CI) Adjusted	*N* = 318 (25%)
*Al Kuwari *et al*. 2020 (Qatar)*	Cross-sectional study Community study Inclusion: 28 February to 18 April 2020	10	Inclusion: retrospective identification of all confirmed cases of COVID-19 infection between 28 February and 18 April 2020	RT-PCR test	*Severe or critical illness* Severity of illness at the time of presentation was determined by expert coders using criteria suggested by the WHO, including admission to acute care or ICU, need for mechanical ventilation, oxygen saturation and supplemental oxygen requirement^2^	*N* = 5,685 Age range 0- > 60; median age (IQR): 34 (28–43); females: 11.1%; nationality: Indian (27.4%), Bangladeshi (18.9%), Nepalese (18.4%), Qatari (8.7%), Pakistani (6.2%), Filipino (3.3%), Egyptian (3.1%), Sri Lankan (1.9%), Sudanese (1.6%), others (10.3%)	Measured by disease count; 9 conditions retrieved from electronic medical records coded using ICD-10-AM *N* conditions = *N* (%) population 0 = 4,753 (83.6) 1 = 384 (6.8) 2 = 139 (2.5) 3 = 121 (2.1) ≥ 4 = 53 (0.9) Missing 235 (4.1) ≥ 2 conditions = 313 (5.7)	Hypertension, diabetes mellitus, cardiovascular disease, chronic lung disease, chronic kidney disease, solid organ malignancy, tuberculosis, chronic liver disease, autoimmune disease	Frailty not assessed	OR (95% CI) Adjusted	Severe illness *N* = 82 (1.4%) Critical illness *N* = 35 (0.6%)
*Cardoso *et al*. 2020 (Portugal)*	Cohort Community study Inclusion: 2 March to 21 April 2020	8	Median (IQR) follow-up: 27 (19–33) days	RT-PCR test	*ICU admission or mortality* Composite of ICU admission or all-cause mortality of confirmed cases of SARS-CoV-2 infection	*N* = 18,647 Median age (IQR): 50 (36–66); females: 58.7%	Measured by disease count; 8 conditions considered, source of information not specified; *N* conditions = N (%) participants 0 = 15,651 (83.9) 1 = 2,213 (11.9) 2 = 600 (3.2) ≥ 3 = 183(1) ≥ 2 conditions = 783 (4.2)	Diabetes mellitus, respiratory, neurological/muscular, malignancy, cardiovascular/kidney, haematological, liver, HIV infection	Frailty not assessed	OR (95% CI) Unadjusted and adjusted	ICU admission or death *N* = 687 (3.7%)
*Khan *et al*. 2020 (Saudi Arabia)*	Retrospective cohort COVID-19 confirmed cases located in health care facilities Inclusion: 1 March to 31 March 2020	8	Follow-up: 1 March to 31 March 2020	RT-PCR test	*ICU admission or mortality* ICU admission or death among COVID-19 patients	*N* = 648 Age range (1- > 60); median age (IQR): 34 (19); females: 47.2%; occupation: working in health care facilities 101 (15.6%); military 21 (3.2%); others 526 (81.2%)	Measured by disease count; 7 conditions retrieved from electronic medical records coded using ICD-10 *N* conditions = *N* (%) participants 0 = 382 (59) 1 = 188 (29) ≥ 2 = 78 (12)	Diabetes mellitus, hypertension, cardiac diseases, chronic respiratory diseases, cancer, immunodeficiency and chronic kidney diseases	Frailty not assessed	OR (95% CI) Unadjusted and adjusted	ICU admission or death *N* = 77 (11.9%)

95% CI 95% confidence interval, *CCI* Charlson Comorbidity Index, *COPD* chronic obstructive pulmonary disorder, *HFRS* hospital frailty risk score, *HR* hazard ratio, *ICD-10* International Classification of Diseases 10th edition, *ICU* intensive care unit, *OR* odds ratio, *RT-PCR* real-time polymerase chain reaction test, *HR* hazard ratio, *SARI* severe acute respiratory infection, *WHODAS* World Health Organisation Disability Assessment Schedule 2.0-12L

^a^Cohort studies: maximum score = 9; cross-sectional studies: maximum score = 10

^b^Two outcomes assessed

^1^Defined based on the National Council for the Evaluation of Social Policy (CONEVAL)

^2^World Health Organisation definitions of severity of illness in persons with COVID-19 infection (bmjopen-2020-040428supp002_data_supplement.pdf)

None of the studies reported the long-term health outcomes. Mortality was the most frequently studied outcome. In most studies, adjusted associations with age and sex were the preferred adjustment factors. The largest study included 24,367,476 individuals compared with 308 in the smallest. Table 1 presents the study characteristics.

#### Etiological role of multimorbidity

The two retained studies [36, 37] were cohorts with large study samples conducted in community settings. Disease burden was measured using the Charlson Comorbidity Index (CCI) (Table 1). Both studies showed a similar direction of the association, namely that the likelihood of poor outcomes increased with a higher CCI score. With each increase in CCI score, the odds ratio (OR) (95% CI) was 1.30 (1.28–1.32) for infection, 1.47 (1.44–1.50) for hospitalisation and 1.53 (1.48–1.59) for mortality [36], and 1.09 (1.06–1.13) and 1.08 (1.03–1.14) for CCI > 0 for hospitalisation and mortality (37), respectively (Table 2).

**Table 2:** Associations between multimorbidity and COVID-19 outcomes among population-representative samples (etiological and prognostic role)

Etiological role						
First author (Country)	Sample	Outcome	*N* (%) of people who experienced the outcome (total and per multimorbidity level where available)	Association with multimorbidity Unadjusted	Association with multimorbidity Adjusted	Adjustment factors
*Mak *et al*. 2021 (England)*	Community study *N* = 410,199	SARS-CoV-2 infection	*N* = 7,590 (1.85%)	NA	OR (95% CI) for 1 CCI score increase = 1.30 (1.28–1.32)	Age and sex
		Hospitalisation for Covid-19	*N* = 2,812 (0.69%)	NA	OR (95%CI) for 1 CCI score increase = 1.47 (1.44–1.50)	Age and sex
		Mortality	*N* = 514 (0.1%)	NA	OR (95% CI) for 1 CCI score increase = 1.53 (1.48–1.59)	Age and sex
*Izurieta *et al*. 2020 (US)*	Community study *N* = 24,367,476	SARS-CoV-2 infection	NA	NA	NA	NA
		Hospitalisation for Covid-19	*N* = 27,961 (0.11%)	NA	OR (95% CI) for CCI > 0 = 1.09 (1.06–1.13)	Sex, aged into Medicare, race, area deprivation index, Covid-19 circulation rate, population density by county, influenza vaccination status, presence of individual medical conditions, frailty conditions, immunocompromised status
		Mortality	*N* = 12,613 (0.05%)	NA	OR (95% CI) for CCI > 0 = 1.08 (1.03–1.14)	Sex, aged into Medicare, race, area deprivation index, Covid-19 circulation rate, population density by county, influenza vaccination status, presence of individual medical conditions, frailty conditions, immunocompromised status
Prognostic role						
*Haase *et al*. 2020 (Denmark)*	COVID-19 ICU patients *N* = 308	Mortality	*N* = 118 (37%)	*N* conditions = mortality rate (95% CI) 0 = 0.24 (0.16–0.35) 1 = 0.36 (0.26–0.48) 2 = 0.46 (0.35–0.58) 3 = 0.5 (0.33–0.66) 4 = 0.4 (0.12–0.74) 5 = 0.83 (0.36–1) 6 = 1	NA	NA
*Millán-Guerrero *et al*. 2020 (Mexico)*	Patients attending any health care facility and diagnosed with COVID-19 *N* = 231,772	Mortality	*N* = 28,510 (12.3%)	*N* conditions = HR (95% CI) 0 = 1 1 = 1.48 (1.44–1.52) 2 = 2.14 (2.07–2.21) 3 = 2.57 (2.46–2.69) ≥ 4 = 3.05 (2.84–3.27)	*N* conditions = HR (95% CI) 0 = 1 1 = 1.19 (1.16–1.23) 2 = 1.43 (1.39–1.48) 3 = 1.57 (1.50–1.65) ≥ 4 = 1.72 (1.60–1.84)	Age, sex, poverty level^1^
*Reilev *et al*. 2020 (Denmark)*	SARS-CoV-2 PCR-positive cases (80% community-managed and 20% hospitalised) *N* = 11,122	Mortality	*N* = 577 (5.2%) *N* conditions = N (%) deaths 0 = 30 (5.2) 1 = 92 (16) 2 = 108 (19) 3 = 122 (21) ≥ 4 = 225 (39)	*N* conditions = OR (95% CI) 0 = 1 1 = 7.8 (5.1–11.8) 2 = 20.9 (13.9–31.6) 3 = 42.9 (28.5–64.6) ≥ 4 = 79.0 (53.5–116.7)	*N* conditions = OR (95% CI) 0 = 1 1 = 2.6 (1.6–4.0) 2 = 2.6 (1.7–4.1) 3 = 3.5 (2.2–5.4) ≥ 4 = 5.2 (3.4–8.0)	Age and sex
*Sousa *et al*. 2020 (Brasil)*	Population admitted to hospital or paediatric health care unit *N* = 4,784 (2,570 Covid-19 positive patients; remaining, other SARI patients)	Mortality	*N* = 353 (15.2%)	*N* conditions = OR (95% CI) 0 = 1 1 = 3.01 (2.32–3.91) ≥ 2 = 4.76 (3.45–6.56)	*N* conditions = OR (95% CI) 0 = 1 1 = 3.03 (2.34–3.94) ≥ 2 = 4.81 (3.48–6.63)	Age and sex
*Argoty-Pantoja *et al*. 2021 (Mexico)*	Outpatients and hospitalised COVID-19 patients *N* = 412,017	Mortality	*N* = 45,754 (11.1%)	NA	Outpatients Disease combinations = HR (95% CI) Obesity & hypertension = 2.84 (2.29–3.51) Diabetes & hypertension = 3.58 (3.05–4.22)^2^ Diabetes & obesity = 4.69 (3.53–6.23)^2^ Diabetes & obesity & hypertension = 5.57 (4.54–6.84)^2^ Hospitalised Disease combinations = HR (95% CI) Obesity & hypertension = 1.31 (1.21–1.42) Diabetes & hypertension = 1.51 (1.43–1.59)^2^ Diabetes & obesity = 1.32 (1.18–1.46) Diabetes & obesity & hypertension = 1.66 (1.54–1.79)^2^	Indigenous population, age, sex, COPD, chronic kidney disease
*Hernandez-Vasquez *et al*. 2020 (Mexico)*	Community *N* = 51,053	Mortality	*N* = 5,233 (10.3%)	*N* conditions = OR (95% CI) 0 = 1 1 = 2.57 (2.39–2.77) 2 = 4.40 (4.06–4.77) ≥ 3 = 6.56 (5.96–7.21)	*N* conditions = OR (95% CI) 0 = 1 1 = 1.89 (1.75–2.04) 2 = 2.51 (2.30–2.73) ≥ 3 = 3.49 (3.15–3.86)	Age, sex, smoking status
*Cho *et al*. 2020 (South Korea)*	Community *N* = 7,327	Mortality	*N* = 223 (3%)	HR (95% CI) for CCI score increase 1.35 (1.30–1.39)	HR (95% CI) for CCI score increase 1.14 (1.09–1.20)	Age, sex, hypertension
*Mak *et al*. 2021 (England)*	COVID-19 inpatients *N* = 2,812	Mortality	*N* = 417 (14.8%)	NA	OR (95% CI) for 1 CCI score increase = 1.17 (1.11–1.23)	Age and sex
*Navaratnam *et al*. 2021 (England)*	COVID-19 inpatients *N* = 79,124	Mortality	*N* = 28,200 (30.8%)	NA	OR (95% CI) associated with CCI: 0 = 1.0 1 = 1.60 (1.51–1.68) 2 = 2.06 (1.94–2.18) 3 = 2.41 (2.27–2.57) ≥ 4 = 3.04 (2.88–3.22)	Age, sex, deprivation index, ethnicity, date of discharge
*Ticinesi *et al*. 2021 (Italy)*	COVID-19 inpatients *N* = 1,264	Mortality	*N* = 318 (25%) N deaths according to multimorbidity (no/yes): No: 40 (12%) Yes: 277 (30%)	NA	OR (95% CI) associated with multimorbidity (binary): 0 = 1.0 ≥ 2 = 1.64 (1.10–2.45) OR (95% CI) associated with number of diseases: 1.17 (1.04–1.31)	Adjustment factors for association with multimorbidity (as a binary variable): age, sex, period of admission Adjustment factors for association with disease count: age classes, PaO2/FiO2 on admission, RT-PCR test positive on admission, admission after 3/23/2020, CT visual score, lymphocyte count, platelet count, creatinine, lactate dehydrogenase, white blood cell count, total dependency in daily activities
*Reilev *et al*. 2020 (Denmark)*	SARS-CoV-2 PCR-positive cases (80% community-managed and 20% hospitalised) *N* = 11,122	Hospitalisation	*N* = 2,254 (20%) N conditions = *N* (%) hospitalisations 0 = 502 (22) 1 = 484 (21) 2 = 398 (18) 3 = 364 (16) ≥ 4 = 506 (22)	*N* conditions = OR (95% CI) 0 = 1 1 = 2.7 (2.4–3.1) 2 = 5.9 (5.1–6.9) 3 = 12.3 (10.3–14.6) ≥ 4 = 19.3 (16.3–22.9)	*N* conditions = OR (95% CI) 0 = 1 1 = 1.7 (1.5–2.0) 2 = 2.1 (1.8–2.5) 3 = 3.1 (2.5–3.8) ≥ 4 = 3.9 (3.2–4.8)	Age and sex
*Cho *et al*. 2020 (South Korea)*	Community study *N* = 7,327	Mechanical ventilation	*N* = 123 (1.7%)	OR (95% CI) per CCI score 1.34 (1.27–1.42)	OR (95% CI) per CCI score 1.10 (1.01–1.18)	Age, sex, hypertension
*Al Kuwari *et al*. 2020 (Qatar)*	Community study *N* = 5,685	Severe or critical illness	Severe illness *N* = 82 (1.4%) Critical illness *N* = 35 (0.6%) *N* conditions = *N* (%) severe or critical illness 0 = 67 (1.4) 1 = 28 (7.3) ≥ 2 = 22 (7.1)	NA	*N* conditions = OR (95% CI) 0 = 1 1 = 5.43 (3.41–8.63) ≥ 3^a^ = 6.16 (3.35–11.32)	Age, sex, nationality (Qatari vs non-Qatari)
*Cardoso *et al*. 2020 (Portugal)*	Community study *N* = 18,647	ICU admission or mortality	ICU admission or death *N* = 687 (3.7%) *N* conditions = N (%) ICU admission or death 0 = 298 (43.4) 1 = 233 (33.9) 2 = 103 (15.0) ≥ 3 = 53 (7.7)	*N* conditions = OR (95% CI) 0 = 1 1 = 6.06 (5.08–7.24) 2 = 10.68 (8.39–13.59) ≥ 3 = 21.00 (14.96–29.49)	*N* conditions = OR (95% CI) 0 = 1 1 = 2.95 (2.45–3.56) 2 = 3.57 (2.77–4.60) ≥ 3 = 6.00 (4.21–8.57)	Age and sex
*Khan *et al*. 2020 (Saudi Arabia)*	COVID-19-confirmed cases located in health care facilities *N* = 648	ICU admission or mortality	ICU admission or death *N* = 77 (11.9%)	*N* conditions = OR (95% CI) ≥ 1 = 2.01 (1.24–3.28) ≥ 2 = 3.38 (1.91–5.99)	*N* conditions = OR (95% CI) ≥ 1 = 1.51 (0.87–2.62) ≥ 2 = 2.57 (1.33–4.97)	Age and sex

95% CI 95% confidence interval, *CCI* Charlson Comorbidity Index, *HR* hazard ratio, *ICU* intensive care unit, *NA* not available, *OR* odds ratio, *PCR* polymerase chain reaction test, *SARI* severe acute respiratory infection

^a^as reported by authors

^1^Defined by the patient’s municipality of residence using CONEVAL data (25)

^2^Interaction with time

#### Prognostic role of multimorbidity

##### Studies reporting on mortality

Six cohorts and four cross-sectional studies reported on mortality [36, 38, 39, 41, 43, 45–49]. Study samples ranged from 308 to 412,017 participants and covered all age groups. The proportion of females was between 26 and 60%.

Multimorbidity burden was usually based on disease count (six studies), while three studies used CCI and one explored combinations of diseases (dyads or triads composed of diabetes, obesity and hypertension). The number of conditions ranged from 7 to 23, and the data were mainly retrieved from medical records or administrative data (Table 1).

Regardless of the multimorbidity measure, the studies demonstrated an increasing likelihood of mortality with an increasing disease burden. The direction and strength of the association were rather consistent across studies with a significant dose–response relationship. Of the six studies with multimorbidity based on the disease count, two reported multimorbidity as a binary variable (0, 1, ≥ 2), while four studies presented the association for more than two diseases, including one with mortality rate, one with the hazard ratio (HR) and two with OR (Table 2). Odds Ratio for the association between two diseases and mortality in the adjusted models was similar OR (95% CI) of 2.6 (1.7–4.1) [47] and OR (95% CI) 2.51 (2.3–2.7) [43]. Both studies were adjusted for age and sex, while that by Hernández-Vásquez et al. [43] were additionally adjusted for smoking status. The studies also presented unadjusted models with stronger associations; the difference between the unadjusted and adjusted models was particularly high in the study of Reilev et al. [47 with OR (95% CI) of 79.0 (53.5–116.7) for four or more diseases in the unadjusted model versus 5.2 (3.4–8.0) in the adjusted model. Two studies with a continuous CCI score estimated the association with mortality with HR (95% CI) of 1.14 (1.09–1.20) [49] and OR (95% CI) of 1.17 (1.11–1.23) [36]. In the study of Argoty-Pantoja et al. [41], the disease triad of “diabetes & obesity & hypertension” was associated with the highest likelihood of death compared with dyads of these three conditions, with HR (95% CI) of 5.57 (4.54–6.84) and 1.66 (1.54–1.79) among outpatients and hospitalised patients, respectively (Table 2).

##### Studies reporting on hospitalisation

Only one study reported on hospitalisation [47]. This was a cohort study with 80% community managed cases and 20% hospitalised cases. Disease burden was measured based on disease count (17 conditions). Information on patient health status was retrieved from the administrative and health registries (Table 1). The adjusted risk of hospitalisation increased with the increasing disease count (e.g., for four or more diseases, OR (95% CI) = 3.9 (3.2–4.8)) (Table 2).

##### Studies reporting on mechanical ventilation

One population-based cohort study reported on mechanical ventilation [49]. The study included 7,327 participants (60% women) with a mean age of 47 ± 19 years. Information on health status was retrieved from administrative data (Table 1). Overall, 1.7% of the total study sample received mechanical ventilation (Table 2). The likelihood of mechanical ventilation increased with increasing CCI score (OR (95% CI) = 1.10 (1.01–1.18)).

##### Studies reporting on critical or severe illness

One study observed the association between multiple chronic conditions and severe or critical illness (defined based on the WHO guidelines) [40]. A total of 5,685 participants were included in the analysis, with 11% females and a median age (IQR) of 34 (28–43) years. Disease burden was estimated based on disease count (nine chronic conditions) retrieved from electronic medical records. Overall, 1.4% of the population was severely ill, while 0.6% was critically ill (Table 1). Among severely or critically ill patients, 7.1% had two or more diseases (vs 5.7% in the total population). The likelihood of severe or critical illness was higher in the population with more diseases; for example, for three or more diseases versus one disease, OR (95% CI) was 6.16 (3.35–11.32) and 5.43 (3.41–8.63), respectively (Table 2).

##### Studies reporting on intensive care unit admission or mortality

Two cohort studies reported on ICU admission or mortality [42, 44]. The study of Cardoso et al. [42] had a much larger sample size with 18,647 individuals, a female proportion of 59% and an older population with a median age (IQR) of 50 (36–66) years compared with that of Khan et al. [44] (*n* = 684, 47% female and median age MD (IQR) = 34 (19)) (Table 1). Both studies found higher OR for ICU admission or mortality with an increased disease count: OR (95% CI) of 2.57 (1.33–4.97) for two or more diseases [44] and 3.57 (2.77–4.60) for two diseases [42] (Table 2).

### Frailty

#### Study characteristics

Five studies reporting on frailty corresponded to our research criteria [36, 37, 39, 50, 51]. Two studies described the etiological role of frailty [36, 37] and four its prognostic role [36, 39, 50]. Mak et al. [36] reported on both objectives. Four studies involved cohorts, two of which scored the maximum of 9 points and two 8 points for quality; the cross-sectional study scored 9 points (Table 1 and Supplementary material 5). One study was conducted in a community setting, three in a hospitalised setting and one in the ICU; the study of Mak et al. [36] was conducted in two settings. All studies indicated the applied definition of frailty. Only one study used an individual frailty assessment based on a questionnaire at inclusion, while the others used a frailty index based on electronic medical records. COVID-19 diagnosis was usually confirmed with a PCR test.

Mortality was the most frequently studied outcome. None of the studies reported the long-term outcomes. The largest study had 24,367,476 individuals compared with 160 in the smallest. Table 1 presents the study characteristics.

#### Etiological role of frailty

The two retained studies which explored the etiological role of frailty were cohorts, with large study samples involving 410,199 [36] and 24,367,476 individuals [37]. Frailty was assessed using the Hospital Frailty Risk Score computed with ICD-10 codes from hospital records 2 years before the COVID-19 pandemic [36] or via a claims-based frailty index based on ICD-10 codes from hospital records 6 months prior to the study [37] (Table 1). For both studies, the likelihood of poor outcomes increased with a higher probability of frailty: for example, OR (95% CI) associated with an intermediate and high frailty risk were respectively 2.23 (2.03–2.45) and 9.02 (8.10–10.04) for infection, 3.84 (3.41–4.31) and 15.25 (13.45–17.30) for hospitalisation and 5.17 (4.09–6.52) and 20.40 (16.24–25.63) for mortality [36]. OR (95% CI) associated with a 10% increase in the frailty probability was 1.47 (1.45–1.49) for hospitalisation and 1.58 (1.55–1.60) for mortality [37] (Table 3).

**Table 3:** Associations between frailty and COVID-19 outcomes among population-representative samples (etiological and prognostic role)

Etiological role						
First author (Country)	Sample	Outcome	*N* (%) of people who experienced the outcome (total and per frailty level where available)	Association with frailty Unadjusted	Association with frailty Adjusted	Adjustment factors
*Mak *et al*. 2021 (England)*	General population *N* = 410,199	SARS-CoV-2 infection	*N* = 7,590 (1.85%)	NA	OR (95% CI) associated HFRS bands: Low risk = 1 Intermediate risk = 2.23 (2.03–2.45)^a^ High risk = 9.02 (8.10–10.04)^a^	Age and sex
		Hospitalisation for Covid-19	*N* = 2,812 (0.69%)	NA	OR (95% CI) associated HFRS bands: Low risk = 1 Intermediate risk = 3.84 (3.41–4.31)^a^ High risk = 15.25 (13.45–17.30)^a^	Age and sex
		Mortality	*N* = 514 (0.1%)	NA	OR (95% CI) associated HFRS bands Low risk = 1.0 Intermediate risk = 5.17 (4.09–6.52)^b^ High risk = 20.40 (16.24–25.63)^b^	Age and sex
*Izurieta *et al*. 2020 (US)*	General population *N* = 24,367,476	Hospitalisation for Covid-19	*N* = 27,961 (0.11%)	NA	OR (95% CI) for 10% increase in frailty probability = 1.47 (1.45–1.49)	Sex, aged into Medicare, area deprivation index, Covid-19 circulation rate, population density by county, influenza vaccination status, presence of numerous medical conditions, immunocompromised status, race
		Mortality	*N* = 12,613 (0.05%)	NA	OR (95% CI) for 10% increase in frailty probability = 1.58 (1.55–1.60)	Sex, aged into Medicare, area deprivation index, Covid-19 circulation rate, population density by county, influenza vaccination status, presence of numerous medical conditions, immunocompromised status, race
Prognostic role						
*Mak *et al*. 2021 (England)*	COVID-19 inpatients *N* = 2,812	Mortality	*N* = 417 (14.8%)	NA	OR (95% CI) associated HFRS bands Low risk = 1 Intermediate risk = 1.53 (1.13–2.05)** High risk = 1.41 (1.04–1.90)^b^	Age and sex
*Navaratnam *et al*. 2021 (England)*	COVID-19 inpatients *N* = 79,124	Mortality	*N* = 28,200 (30.8%)	NA	OR (95% CI) associated HFRS bands None: 1.0 Mild: 0.78 (0.71 – 0.85) Moderate: 1.51 (1.41–1.63) Severe: 1.80 (1.67 – 1.94)	Age, sex, deprivation index, ethnicity, date of discharge
*Hodgson *et al*. 2021 (Australia)*	COVID-19 patients admitted to ICU *N* = 160	Mortality or new disability	*N* = 85 (53.1%)	NA	OR (95% CI) associated CFS = 1.49 (1.05 – 2.11), p = 0.025	APACHE II (acute physiology and chronic health evaluation)
*Kundi *et al*. 2020 (Turkey)*	All COVID-19 hospitalised patients (≥ 65 years) *N* = 18,234	All-cause mortality	Overall *N* = 3,315 (18.2%) Low HFRS (< 5): *N* = 697 (12.0%) Intermediate HFRS (5–15): *N* = 1,751 (18.2%) High HFRS (> 15): *N* = 867 (31.0%)	NA	OR (95% CI) associated HFRS bands Low risk: 1.00 (ref) Intermediate risk: 1.482 (1.334–1.646) High risk: 2.084 (1.799–2.413)	Age, sex, comorbidities
		ICU	Overall *N* = 4,510 (24.7%) Low HFRS (< 5): *N* = 975 (16.8%) Intermediate HFRS (5–15): *N* = 2,397 (24.9%) High HFRS (> 15): *N* = 1,138 (40.6%)	NA	OR (95% CI) associated HFRS bands Low risk: 1.00 (ref) Intermediate risk: 1.460 (1.334–1.598) High risk: 2.221 (1.951–2.527)	Age, sex, comorbidities
		Invasive mechanical ventilation	Overall *N* = 3,080 (16.9%) Low HFRS (< 5): *N* = 650 (11.2%) Intermediate HFRS (5–15): *N* = 1,653 (17.2%) High HFRS (> 15): *N* = 777 (27.7%)	NA	OR (95% CI) associated HFRS bands Low risk: 1.00 (ref) Intermediate risk: 1.376 (1.240–1.527) High risk: 1.769 (1.531–2.046)	Age, sex, comorbidities

95% CI 95% confidence interval, *HFRS* hospital frailty risk score, *CFS* Clinical frailty scale, *ICU* intensive care unit, *NA* not available, *OR* odds ratio

^a^Significant with a false discovery rate corrected significance level at 0.048

^b^Significant with a false discovery rate corrected significance level at 0.032

#### Prognostic role of frailty

Of the studies investigating the prognostic role of frailty, three were cohort studies (study samples *n* = 2,812 [36], *n* = 91,154 [39] and *n* = 160 [50]), while one was cross-sectional (study sample *n* = 18,234 [51]). In Navaratnam et al. [39], 34.8% of the population was aged over 80 years. The population in the study of Mak et al. [36] had a mean age (SD) of 69 (± 8.7) years, which was slightly older than in Hodgson et al. [50] (62 years (IQR: 55–71)), which also had the smallest proportion of females (39.4%). Kundi et al. [51] had the highest proportion of females (53.4%) with a mean population age (SD) of 74 (± 7.4) years (Table 1).

Frailty was assessed using the Hospital Frailty Risk Score [36, 39, 51] or the Clinical Frailty Scale at the time of ICU admission [50] (Table 1).

All studies reported an increased risk of mortality, or mortality or new disability with increasing frailty scores. Three studies that used the Hospital Frailty Risk Score reported similar risks for mortality: OR (95% CI) of 1.53 (1.13–2.05) [36], 1.48 (1.33–1.65) [51] and 1.51 (1.41–1.63) [39] for intermediate or moderate mortality risks. Reported high or severe risks for mortality differed more substantially between the studies: i.e., OR (95% CI) 1.41 (1.04–1.90) [36], 2.08 (1.80–2.41) [51] and 1.80 (1.67–1.94) [39]. All three studies adjusted for sex and age in their models, although Navaratnam et al. [39] adjusted additionally for the deprivation index, ethnicity and date of discharge, and Kundi et al. [51] also for comorbidities, which may be considered to be an overadjustment. In addition, Kundi et al. [51] reported an increased likelihood for ICU admission and invasive mechanical ventilation: for example, OR (95% CI) of 1.38 (1.24–1.53) and 1.77 (1.53–2.05) for invasive mechanical ventilation with intermediate- and high-risk scores, respectively (Table 2).

## Discussion

### Summary and discussion of the findings

This systematic review adopted a population-based approach and investigated the etiological and prognostic roles of multimorbidity and frailty in terms of COVID-19 health outcomes in the early years of the pandemic. Literature was scarce, especially for frailty, and the studies focused on short-term outcomes, mostly reporting on mortality. An increased risk of poorer outcomes was associated with higher multimorbidity or frailty levels, which was observed across all measurements of multimorbidity and frailty. We did not identify any studies examining the long-term outcomes.

To account for multiple conditions, researchers most commonly used disease count and Charlson comorbidity index. Only one study examined the association between disease combinations and COVID-19 outcomes. However, it was limited to three cardiometabolic conditions and thus cannot be considered representative of the population with multimorbidity, although it was retained based on the cut-off point of two or more conditions for multimorbidity to emphasise the pertinence of exploring the effects of disease combinations. Identifying the most frequent and most harmful combinations is essential, as the joint effect of disease patterns may be stronger than their individual additive effects [52]. Despite the broad body of evidence on the association between individual chronic conditions and COVID-19-related outcomes [18], studies on patients with multiple conditions are clearly less numerous, particularly among population-representative samples. Among the 14 studies included in this review, only two focused on multimorbidity by specifying the multimorbidity definitions they used.

Similarly, all studies on frailty in our review reported a significant association between higher frailty scores and poorer outcomes. While population-representative studies are scarce, there is substantial evidence with smaller hospital-based samples [53, 54]. These studies confirm the strong association between frailty levels and poorer short-term outcomes such as mortality, although they may underestimate the strength of the association, as they are based on severe cases. The study of Mak et al. [36], which provided estimates at the population level and among hospitalised patients, clearly demonstrated a stronger association between frailty and mortality at the population level. The main constraint of the evidence provided by this review regarding frailty relates to the fact that most included studies used electronic frailty scores. These scores are not based on face-to-face examination nor on assessment of individuals' functional and cognitive performances. This information would improve the accuracy and sensitivity of frailty measurement and provide more robust estimations of associations with COVID-19 outcomes. In addition, the identification of frailty at the primary care level could improve prevention in this population during this or similar public health emergencies in the future. There is still a lack of well-performed population-based studies that assess the actual association between frailty in the community-based population and COVID-19 severity.

Our results present the state of the evidence in the early years of the COVID-19 pandemic. Since our review, several studies on population representative samples have been released for multimorbidity [55–62] and frailty [63–65]. They also consider earlier waves of the pandemic (study period between January 2020 and July 2021) and address short-term outcomes, notably in terms of infection, hospitalisation, ICU admission and mortality. The studies support our findings underlying the higher risk of poorer outcomes with the higher number of diseases or with a CCI score increment [55, 56, 58, 59, 61, 62], with increasing frailty [63–65] and for certain disease patterns such as cardiometabolic or cardiovascular patterns, which presented a stronger association with infection or infection severity [57, 60]. Regarding the risk of infection, however, the findings seem to be less conclusive, for e.g., Catalano et al. [58] showed the lesser likelihood of infection for patients with multimorbidity, even though they were tested more often, which may potentially be explained by their better compliance with the restrictive measures; nevertheless, in the same study, the risk of hospitalisation, ICU admission or death was higher for patients with multimorbidity. The number of emerging studies on population representative samples seems encouraging, although it is clear that making this evidence available requires time.

### Study limitations

Only quantitative studies were considered. As case and qualitative study designs require different approaches for evidence synthesis [66–68], they were excluded for feasibility reasons. Further, we only included scientific publications in English, which may have led to the omission of population-based studies published in other languages. Despite a pilot test being conducted prior to the study and regular weekly meetings to reduce heterogeneity during the screening process [23], disagreements between two reviewers were frequent during the full-text reading phase. However, the quality check was ensured through regular consultations with the third reviewer. The instruments used to assess multimorbidity and frailty varied across the studies, thus making comparisons difficult. This along with other variations such as the insufficient number of studies using the same outcome and the association metrics precluded the possibility of performing meta-analysis, which required a minimum of four comparable studies as indicated in the protocol of the study published earlier [23]. The number and type of diseases also differed across studies, although the most prevalent chronic conditions were included. The use of a medical coding system such as the International Classification of Diseases (ICD) was often not reported. In addition, the study settings differed across the studies (e.g., community setting, hospital setting, ICU), although the population representativeness of the sample was always required with the inclusion of all community or hospitalised cases, for instance. In this regard, studies undertaken in the same setting may be more comparable. The population representativeness of the sample was difficult to determine on several occasions during the screening process, which may have led to the omission of some evidence. The studies most frequently controlled for age and sex, although factors such as poverty level, ethnicity, influenza vaccination status or others were occasionally considered. Where unadjusted models were also presented, the adjusted models showed a less strong association between the risk factors and outcomes, thus indicating that sociodemographic, socioeconomic, biological or behavioural factors may also influence these associations. Certain studies adjusted for individual diseases or frailty in addition to multimorbidity, thus leading to overadjustments and minimising the strengths of the association in the resulting models.

### Recommendations for research and policy

Repeated exposure, different virus variants and vaccination in the later years of the pandemic may have changed the landscape of infection. The intention of our work was to underline the relevance of estimations at the population level to guide public health decisions; here we provide the existence of those estimations for discussed health groups during the initial phases of the pandemic. The studies which discuss the later waves of SARS-CoV-2 infection will be relevant in observing the potential evolution of the associations presented here and in comparing their direction and strength with those of the later waves, while taking into consideration more recent virus variants and the effects of vaccination. Considering that the circulation of variants and vaccination programs differed across geographical regions, recent population-representative studies performed in the same regions and the same settings as those discussed here would be particularly valuable to compare the findings. Researchers should be encouraged to adjust for age and sex at a minimum. As poor socioeconomic characteristics also influence multimorbidity and frailty [52, 69–71] as well as COVID-19 outcomes [72], they should also be considered in models to provide more precise estimates. The majority of the studies identified in our review were conducted in high-income countries, with only a few being performed in middle-income settings. Bearing in mind that life expectancy is increasing worldwide and even at a faster rate in less-off parts of the world [73], given the challenges posed by ageing societies, low-income countries, which endured the most devastating effects of the COVID-19 pandemic, should be urged to estimate the relationships investigated here. Multimorbidity manifests differently across age and sex groups [74], as appears to be the case for the COVID-19 outcomes [11, 75]. The studies identified in our review did not provide stratified estimates for these groups. Observing the associations between multimorbidity or frailty and COVID-19 outcomes separately for men and women and for different age groups would provide more detailed information about the effects of the pandemic.

Unfortunately, no studies on the long-term impacts were identified. Considering the uncertainty of the COVID-19 pandemic during the early years, the concerns about short-term outcomes seems reasonable. It is nevertheless increasingly obvious that the COVID-19 pandemic has left long-lasting consequences on mental health [8], quality of life [76], daily functioning and work capacities [77]. As these consequences risk overburdening the national health and economic systems in the future, they should be recognized and managed in a timely manner.

Lastly, biological mechanisms such as the weakened physiological capacities of multiple organ systems in patients with frailty and/or multimorbidity certainly play an important role in the association with COVID-19 outcomes. Altered biological functioning may increase susceptibility to infection and the likelihood of poorer outcomes. These mechanisms and their potential interaction with COVID-19 pathophysiology merit more research.

## Conclusion

This review provides clear, coherent but limited evidence on the association between increased disease and frailty burden and poorer COVID-19 outcomes in population-representative samples for the early years of the pandemic. Future studies should use the same tools for exposure and outcomes to the feasible extent, to ensure better comparability and certainty in the strength of the associations.

In ageing societies, multimorbidity and frailty represent growing challenges in both developed and developing contexts. Single disease-oriented healthcare systems are already struggling to meet the health and financial demands caused by these complex conditions, and overlooking their importance in the current context risks compounding these issues with the consequences of infectious diseases. The magnitude of these repercussions should be thoroughly explored to guide adequate public health decisions and reduce the impact of COVID-19. Any lessons drawn now may help in the management of any future health crisis should it emerge.

### Supplementary Information

Below is the link to the electronic supplementary material.

Supplementary file1 Supplementary material 1: Initial search strategy (PDF 1192 KB)

Supplementary file2 Supplementary material 2: Search strategy for the update on frailty (PDF 415 KB)

Supplementary file3 Supplementary material 3: Excluded studies and reasons for exclusion (full-text or report screening phase) (XLSX 47 KB)

Supplementary file4 Supplementary material 4: Study quality assessment for studies on multimorbidity (DOCX 18 KB)

Supplementary file5 Supplementary material 5: Study quality assessment for studies on frailty (DOCX 15 KB)

## Data Availability

This systematic review used only already published data.
