# Supplementary material for: Multimorbidity and frailty are associated with poorer SARS-CoV-2-related outcomes: systematic review of population-based studies
Source: Aging Clin Exp Res. 2024 Feb 14;36(1):40. doi: 10.1007/s40520-023-02685-4 (PMC10866755; doi:10.1007/s40520-023-02685-4)
Supplement: Supplementary file 2 — Supplementary file2 Supplementary material 2: Search strategy for the update on frailty (PDF 415 KB) [file 40520_2023_2685_MOESM2_ESM.pdf]

UPDATE FOR FRAILTY 01022022

WHO COVID-19 Global literature on coronavirus disease strategy

(tw:("cross-sectional" OR "cross sectional" OR "case-control" OR "case control" OR cohort OR longitudinal OR "ecological study" OR "ecological studies" OR "ecological design" OR "ecological designs" OR observational OR "observational study" OR "observational studies" OR "observational design" OR "observational designs" OR "prospective study" OR "prospective studies" OR "prospective design" OR "prospective designs" OR "retrospective study" OR "retrospective studies" OR "retrospective design" OR "retrospective designs" OR "prospective observational study" OR "prospective observational studies" OR "retrospective observational study" OR "retrospective observational studies")) AND (tw:(frailty OR frail)) AND entry_date:([20210407 TO 20220201])

PubMed

Search: #1 AND #2 AND #3 Filters: English, from 2021/4/7 - 3000/12/12

Search: #1 AND #2 AND #3 Filters: from 2021/4/7 - 3000/12/12

Search: #1 AND #2 AND #3

Search: "cross-sectional" OR "cross sectional" OR "case-control" OR "case control" OR cohort OR longitudinal OR "ecological study" OR "ecological studies" OR "ecological design" OR "ecological designs" OR observational OR "observational study" OR "observational studies" OR "observational design" OR "observational designs" OR "prospective study" OR "prospective studies" OR "prospective design" OR "prospective designs" OR "retrospective study" OR "retrospective studies" OR "retrospective design" OR "retrospective designs" OR "prospective observational study" OR "prospective observational studies" OR "retrospective observational study" OR "retrospective observational studies" OR case-control studies[MeSH Terms] OR cohort studies[MeSH Terms] OR cross-sectional studies[MeSH Terms]

Search: frailty OR frail OR frailty[MeSH Terms]

Search: ((((((((((((((((((((("Betacoronavirus"[MeSH Terms] OR "Coronavirus Infections"[MeSH Terms]) OR "COVID-19"[Supplementary Concept]) OR "Coronavirus"[MeSH Terms]) OR "Severe Acute Respiratory Syndrome Coronavirus 2"[Supplementary Concept]) OR "2019nCoV"[All Fields]) OR "betacoronavirus*"[All Fields]) OR "corona virus*"[All Fields]) OR "coronavirus*"[All Fields]) OR "coronovirus*"[All Fields]) OR "CoV"[All Fields]) OR "CoV2"[All Fields]) OR "COVID"[All Fields]) OR (("COVID-19"[Supplementary Concept] OR "COVID-19"[All Fields]) OR "covid19"[All Fields])) OR ((((((("COVID-19"[All Fields] OR "covid 2019"[All Fields]) OR "Severe Acute Respiratory Syndrome Coronavirus 2"[Supplementary Concept]) OR "Severe Acute Respiratory Syndrome Coronavirus 2"[All Fields]) OR "2019 ncov"[All Fields]) OR "SARS CoV 2"[All Fields]) OR "2019nCoV"[All Fields]) OR (("wuhan"[All Fields] AND ("Coronavirus"[MeSH Terms] OR "Coronavirus"[All Fields])) AND (2019/12/1:2019/12/31[Date - Publication] OR 2020/1/1:2020/12/31[Date - Publication])))) OR "HCoV-19"[All Fields]) OR "nCoV"[All Fields]) OR "SARS CoV 2"[All Fields]) OR "SARS2"[All Fields]) OR "SARSCoV"[All Fields]) OR (((("sars virus"[MeSH Terms] OR ("sars"[All Fields] AND "virus"[All Fields])) OR "sars virus"[All Fields]) OR ("sars"[All Fields] AND "CoV"[All Fields])) OR "sars cov"[All Fields])) OR (("Severe Acute Respiratory Syndrome Coronavirus 2"[Supplementary Concept] OR "Severe Acute Respiratory Syndrome Coronavirus 2"[All Fields]) OR "SARS CoV 2"[All Fields])) OR "severe acute respiratory syndrome cov*"[All Fields]) AND (2019/11/17:3000/12/31[Date - Entry] OR 2019/11/17:3000/12/31[Date - Publication]) OR "COVID-19"[MeSH Terms] OR "SARS-Cov-2"[MeSH Terms] OR "SARS CoV-2" OR "SARS-CoV-2" OR SARSCoV2 OR "CoV-2" OR "covid 19" OR covid2019 OR "covid-2019" OR "novel CoV" OR "corona pandemic*" OR "wuhan virus*" OR "CoV 2" OR ((wuhan OR hubei OR huanan) AND ("severe acute respiratory" OR pneumonia*) AND outbreak*)

Embase

('frail elderly'/exp OR 'frail elderly' OR 'frailty'/exp OR 'frailty' OR frail*) AND ('longitudinal study'/exp OR 'longitudinal study' OR 'cross-sectional study'/exp OR 'cross-sectional study' OR longitudinal OR 'cross sectional' OR 'case control study'/exp OR 'case control study' OR 'case control' OR cohort OR 'ecological study' OR 'ecological design' OR 'observational study'/exp OR 'observational study' OR 'retrospective study'/exp OR 'retrospective study' OR 'prospective study'/exp OR 'prospective study' OR 'prospective observational study' OR 'prospective observational studies' OR 'retrospective observational study' OR 'retrospective observational studies' OR 'ecological designs' OR 'prospective studies' OR 'retrospective studies' OR 'observational studies' OR 'ecological studies') AND ('coronavirus disease 2019'/exp OR 'coronavirus disease 2019' OR 'covid 2019' OR 'covid 19' OR covid2019 OR covid19 OR sarscov2 OR 'sars cov 2' OR 'sars cov2' OR 'severe acute respiratory syndrome'/exp OR 'severe acute respiratory syndrome' OR 'wuhan virus' OR 'cov 2' OR cov2 OR coronavirus OR 'corona virus' OR 'novel cov' OR 2019ncov OR '2019 ncov' OR 'corona pandemic' OR betacoronavirus OR cov19 OR 'cov 19' OR 'hcov-19' OR ncov OR sars2) AND [english]/lim AND [07-04-2021]/sd NOT [02-02-2022]/sd

PsycINFO

S1

"cross-sectional" OR "cross sectional" OR "case-control" OR "case control" OR cohort OR longitudinal OR "ecological study" OR "ecological studies" OR "ecological design" OR "ecological designs" OR observational OR "observational study" OR "observational studies" OR "observational design" OR "observational designs" OR "prospective study" OR "prospective studies" OR "prospective design" OR "prospective designs" OR "retrospective study" OR "retrospective studies" OR "retrospective design" OR "retrospective designs" OR "prospective observational study" OR "prospective observational studies" OR "retrospective observational study" OR "retrospective observational studies" OR DE("Longitudinal Studies" OR "Followup studies" OR "Retrospective studies" OR "Cohort analysis")

S2

Frailty OR frail

S3

"covid-19" OR "2019-ncov" OR "sars cov 2" OR "cov-19" OR cov19 OR "cov 19" OR 2019nCoV OR betacoronavirus* OR "corona virus" OR coronavirus* OR coronovirus* OR CoV OR CoV2 OR COVID OR covid19 OR "covid 2019" OR "Severe Acute Respiratory Syndrome Coronavirus 2" OR "2019 ncov" OR (wuhan AND Coronavirus) OR "HCoV-19" OR nCoV OR SARS2 OR SARSCoV OR (sars AND virus) OR "sars virus" OR (sars AND CoV) OR "sars cov" OR "severe acute respiratory syndrome cov" OR "SARS CoV-2" OR "SARS-CoV-2" OR SARSCoV2 OR "CoV-2" OR "covid 19" OR covid2019 OR "covid-2019" OR "novel CoV" OR "corona pandemic" OR "wuhan virus" OR "CoV 2" OR ((wuhan OR hubei OR huanan) AND ("severe acute respiratory" OR pneumonia*) AND outbreak*) OR COVID-19 [DE]

S4

S1 AND S2 AND S3
