# Supplementary material for: Multimorbidity and frailty are associated with poorer SARS-CoV-2-related outcomes: systematic review of population-based studies
Source: Aging Clin Exp Res. 2024 Feb 14;36(1):40. doi: 10.1007/s40520-023-02685-4 (PMC10866755; doi:10.1007/s40520-023-02685-4)
Supplement: Supplementary file 5 — Supplementary file5 Supplementary material 5: Study quality assessment for studies on frailty (DOCX 15 KB) [file 40520_2023_2685_MOESM5_ESM.docx]

# Supplementary material 5

# Study quality assessment – studies on frailty

## Study quality assessment - for cohort studies

| Study | SELECTION | | | | COMPARABILITY | OUTCOME | | | TOTAL |
| --- | --- | --- | --- | --- | --- | --- | --- | --- | --- |
|  | **Representativeness of Exposed Cohort** | **Selection of Non Exposed Cohort** | **Ascertainment of Exposure** | **Demonstration Outcome of Interest Not Present at Start of Study** | **Comparability of Cohorts on the Basis of Design or Analysis** | **Ascertainment of Outcome** | **Adequate Length of Follow Up** | **Adequacy of Follow Up** |  |
| Mak et al. 2021 | 1 | 1 | 1 | 1 | 1 | 1 | 1 | 1 | 8 |
| Hodson et al. 2021 | 1 | 1 | 1 | 1 | 2 | 1 | 1 | 1 | 9 |
| Izurieta et al. 2020 | 1 | 1 | 1 | 1 | 1 | 1 | 1 | 1 | 8 |
| Navaratnam et al. 2021 | 1 | 1 | 1 | 1 | 2 | 1 | 1 | 1 | 9 |

## Study quality assessment - for cross-sectional studies

| Study | SELECTION | | | | COMPARABILITY | OUTCOME | | TOTAL |
| --- | --- | --- | --- | --- | --- | --- | --- | --- |
|  | **Sample Representativeness** | **Sample Size** | **Non-respondents** | **Ascertainment of the Exposure (risk factor)** | **Comparability of Different Outcome Groups on the Basis of Design or Analysis** | **Assessment of Outcome** | **Statistical Test** |  |
| Kundi et al. 2020 | 1 | 1 | 1 | 2 | 1 | 2 | 1 | 9 |
